# Berries, Leaves, and Flowers of Six Hawthorn Species (*Crataegus* L.) as a Source of Compounds with Nutraceutical Potential

**DOI:** 10.3390/molecules29235786

**Published:** 2024-12-07

**Authors:** Natalia Żurek, Michał Świeca, Ireneusz Tomasz Kapusta

**Affiliations:** 1 Department of Food Technology and Human Nutrition, College of Natural Sciences, University of Rzeszow, 4 Zelwerowicza St., 35-601 Rzeszow, Poland; nzurek@ur.edu.pl; 2 Department of Food Chemistry and Biochemistry, University of Life Sciences in Lublin, 8 Skromna St., 20-704 Lublin, Poland; michal.swieca@up.lublin.pl

**Keywords:** hawthorn, nutraceutical, antioxidants, anti-inflammatory, anticancer, anti-diabetic, polyphenolic compounds

## Abstract

Designing new forms of food, food additives, and nutraceuticals is necessary due to the growing needs of consumers, as well as the inflammation of civilization diseases, the prevention and treatment of which can be significantly supported by dietary intervention. For this reason, this study aimed to obtain highly bioactive preparations in the form of powders from the fruits, leaves, and flowers of six species of hawthorn (*Crataegus* L.) using solid phase extraction (SPE). Ultra-performance liquid chromatography analysis (UPLC-PDA-MS/MS) showed a high concentration of phenolic compounds (in the range from 31.50 to 66.06 mg/g), including the highest concentration in hawthorn fruit preparations. Fruit preparations also showed the highest antioxidant activity (through scavenging of O_2_˙^−^ and OH˙ radicals), antidiabetic activity (inhibition of α-amylase and α-glucosidase), and anticancer activity, mainly against colon cancer cells (Caco-2). At the same time, hawthorn flower preparations showed the highest biocompatibility against normal colon cells (CCD841CoN) and anti-inflammatory activity (trypsin inhibition). Correlation and principal component analysis (PCA) showed that the health-promoting potential was most influenced by the content of falavan-3-ols. The above findings provide a basis for the industrial use of the developed preparations, which is in line with the current trend in food technology related to the search for new sources of bioactive compounds and the design of highly bioactive food.

## 1. Introduction

Currently, the food market is focused on developing new products without the addition of chemicals and artificial colors and containing new ingredients with a desired effect on the human body. These products are intended, in addition to meeting the basic nutritional needs of the consumer, to improve health and prevent chronic diseases. In this context, the concepts of functional food and nutraceuticals have appeared, which refer to food products, phytochemicals, or their preparation, and show a documented and beneficial effect on human health [1,2].

New scientific research contributes to the continuous improvement of the market for functional foods, nutraceuticals, and dietary supplements. Currently, this food segment is one of the most dynamically developing branches of the food industry. According to estimates, the global value of this market is to reach over 800 billion dollars by 2028. Currently, the value of the nutraceutical market (including dietary supplements) is estimated at 454.5 billion dollars, of which about 80 billion are plant extracts available in the form of powders, capsules, or tablets [3,4]. These values indicate that consumers are becoming increasingly aware of the role of these products on health, which creates the need to design new forms of food with documented health-promoting activity.

Among the compounds with health-promoting effects, great importance is attributed to plant secondary metabolites, including polyphenolic compounds. Polyphenols are a large group of phytochemical substances that exhibit sensory properties, as well as physiological and biological functions that are beneficial to human health, including antioxidant, anti-inflammatory, anti-allergic, and anti-cancer effects [5]. These compounds have recently gained importance due to the possibility of using them as functional food ingredients, which are used in the production of nutraceuticals. Active substances isolated from the plant matrix, given in the form of food as part of the daily diet, can be a very promising tool in the prevention and support of the treatment of certain disease states [6].

The growing interest in polyphenolic compounds as natural food additives and health promoters has led to deeper studies of many plant species that have been commonly used in folk medicine. Among others, hawthorn (*Crataegus* L.) belongs to this category of plants. Hawthorn, considered one of the oldest pharmaceutical plants, occurs commonly in Western Asia, North America, and Europe. It has been reported so far that hawthorn fruits are dominated by oligomeric procyanidins and their glycosides, while leaves and flowers are dominated by phenolic acids and flavonols [7,8,9,10]. In traditional medicine, decoctions of berries, roots, shoots, and bark have been used to treat gastrointestinal disorders, to treat swelling, to treat insomnia, in inflammation of the mucous membrane of the throat and the mouth, and to treat coughs, bronchitis, and asthma. Currently, the most widely known use of hawthorn flowers and fruits is in the treatment of circulatory system disorders, as an antispasmodic, hypotensive, cardiotonic, and anti-atherosclerotic agent [11,12]. Moreover, many studies have shown that hawthorn extracts also exhibit antioxidant [13], anti-inflammatory [14], antibacterial, antifungal, antiviral, gastroprotective [15], immunostimulatory [16], hypolipidemic [17], antithrombotic [18], hepatoprotective [19,20], anti-aging [21], neuroprotective [22], antidepressant [23], and antiobesity effects [24]. However, preparations or products belonging to the functional food segment enriched with active compounds isolated from hawthorn, with documented health-promoting effects, other than a beneficial effect on the circulatory system, are still scarcely available regarding the food market. Therefore, taking into account the dynamically developing market of health-promoting foods described above, as well as the versatility and high importance of compounds present in hawthorn fruits, leaves, and flowers, their assessment remains a valuable task. However, taking up this challenge may bring beneficial effects for the food industry, especially in the context of discovering and developing new sources of nutraceuticals.

In connection with the above, the aim of this work was to develop preparations from hawthorn fruits, leaves, and flowers with a high content of bioactive compounds. Considering that health-promoting activity may depend on the plant species, morphological parts from six hawthorn species were used for the analyses. Highly bioactive hawthorn preparations obtained by the SPE method were assessed for the content of phenolic compounds using the UPLC-PDA-MS/MS method and physicochemical features typical of powder preparations. In addition, the health-promoting potential of the preparations was assessed by measuring antioxidant, anti-inflammatory, antidiabetic, and anticancer activity, and the safety of use was assessed by assessing biocompatibility with normal human cells. Additionally, in order to fully explain the relationships between the analyzed parameters, Pearson correlation analysis and principal component analysis (PCA) were performed. The obtained results constitute the first comprehensive analysis of the possibilities of using hawthorn morphological parts to design highly bioactive preparations with targeted health-promoting effects. The knowledge obtained will indicate new ways of using hawthorn, including the development of nutraceuticals for the food industry, as well as a basis for the development of dietary supplements and hygiene products in the cosmetics and medical industries.

## 2. Results

### 2.1. Physicochemical Parameters

Extracts available in powder form are the most suitable nutraceutical form of phytochemicals, due to their greater physical and chemical stability in the solid state, as well as the possibility of easy introduction into food products or the preparation of capsules. For nutraceuticals in powder form, it is important to conduct an assessment of physicochemical parameters, which allows for the maintenance of their health benefits during storage as well as in the finished product [25,26].

**Figure 1:**
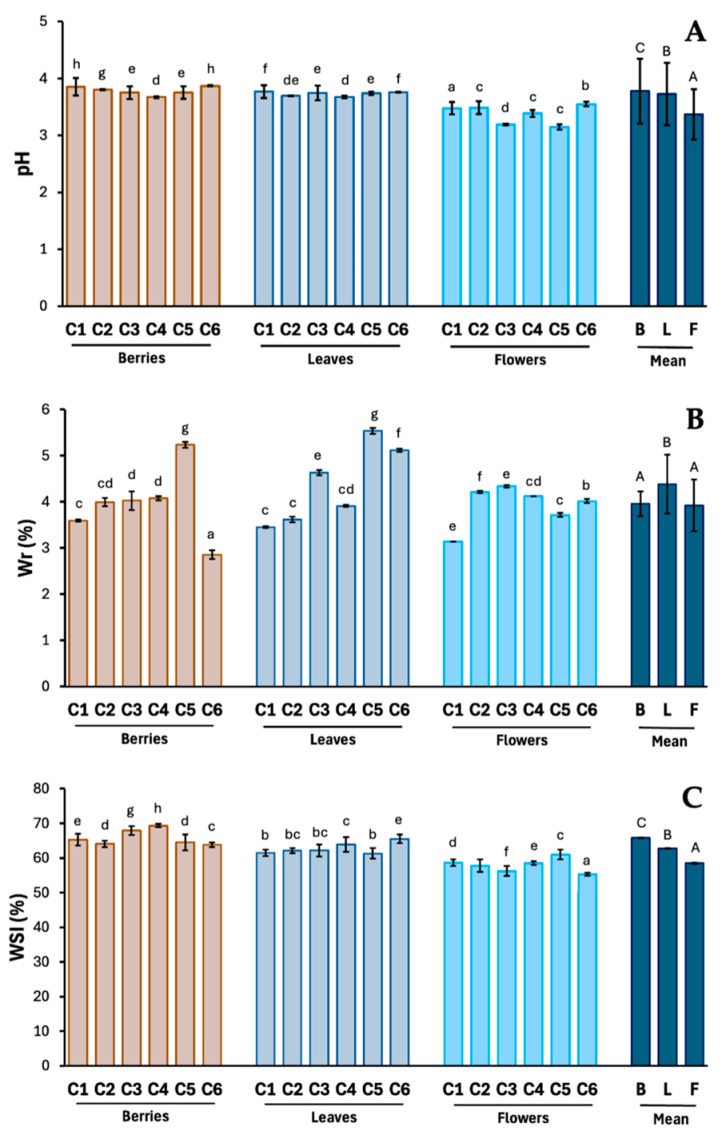
pH value (**A**), equilibrium moisture content (**B**), and water solubility index (**C**) of hawthorn berries, leaves, and flower preparations. Results are expressed as mean (n = 3) ± SD. The values in the columns marked with different letters (lowercase letters, between species and morphological parts; uppercase letters, between means for morphological parts) indicate statistically significant differences (*p* < 0.05). Explanations: C, *Crataegus*; 1–6, hawthorn species (see Section 2.2. Plant material); B, berries; L, leaves; F, flowers.

An important aspect to consider when using phytochemicals as food additives is their stability, which is greatly influenced by the pH value. It has been observed that polyphenolic compounds decompose more rapidly with increasing values for this parameter [25,26]. Polyphenols have been shown to be unstable in neutral and alkaline solutions, decomposing within a few minutes, while remaining stable in acidic conditions [27]. The pH value in previous studies on hawthorn was assessed only for fruits. Alirezalu et al. [28] and Serce et al. [29] obtained values ranging from 3.03 (*C. orientalis*) to 4.35 (*C. curvisepala*). In our own studies, this range for preparations obtained from fruits, leaves, and flowers was at a uniform level from 3.20 (flowers *C. monogyna*) to 3.87 (berries *C. laevigata*), with the highest average value being shown for preparations from fruits and the lowest from flowers (Figure 1). These differences may result from a higher content of organic acids in fruits than in flowers, which was confirmed in other studies [28,29]. Nevertheless, the obtained results are consistent with the data presented above, as well as with the results obtained by other authors for solid polyphenol extracts. Myint et al. [30] assessed the stability of polyphenols isolated from sweet potato and stevia leaves and found that they retained the highest stability in the pH range of 2.0–6.0.

Equilibrium moisture (Wr) is another important property for solid preparations. According to the available literature, powders with Wr ranging from 2.5 to 7.0% are characterized by appropriate flowability, bulk density, particle agglomeration, and better packing [25]. In addition, Wr is extremely important for maintaining the chemical and microbiological stability of preparations. It is believed that a moisture content below 10% is suitable for food powder to prevent mold growth [31]. In our own studies, Wr values of hawthorn preparations ranged from 2.86% (berries *C. laevigata*) to 5.54% (leaves *C. x macrocarpa*), within the recommended range above, which may also indicate their other beneficial physicochemical and functional properties.

The last parameter, the water solubility index (WSI), is a key parameter reflecting the behavior of the product in the water phase and a general criterion for determining the quality of powder reconstitution. The WSI of powders can be influenced by many parameters, both the composition and moisture of a given powder, as well as the method and parameters of the drying process. According to available literature data, this parameter ranges from several to several dozen percent [26,32]. According to the obtained data, the highest WSI value was characteristic of preparations obtained from fruits, and the lowest from flowers. The estimated values ranged from 60.32% (flowers *C. laevigata*) to 69.34% (berries *C. laevigata x rhipidophylla x monogyna*). So far, this parameter has not been assessed for hawthorn powders. However, comparable values were obtained in the works of Sadowska et al. for chokeberry powders [33] and blackcurrant powders [34], showing at the same time that the value of this parameter is shaped by the presence of fibers.

### 2.2. Total Polyphenolic, Flavonoid, and Proanthocyanidin Contents

**Figure 2:**
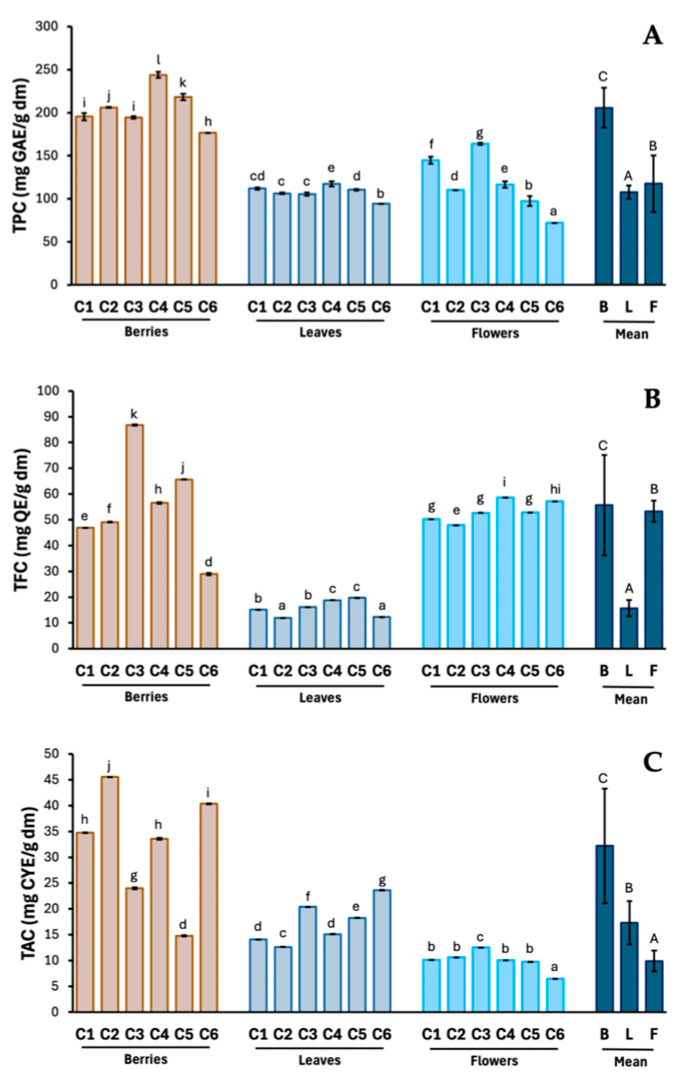
Total content of polyphenols (**A**), flavonoids (**B**), and proanthocyanidins (**C**) in hawthorn berries, leaves, and flower preparations. Results are expressed as mean (n = 3) ± SD. The values in the columns marked with different letters (lowercase letters, between species and morphological parts; uppercase letters, between means for morphological parts) indicate statistically significant differences (*p* < 0.05). Explanations: C, *Crataegus*; 1–6, hawthorn species (see Section 2.2. Plant material); B, berries; L, leaves; F, flowers.

The obtained results indicate that the content of polyphenolic compounds, flavonoids, and total proanthocyanidins is significantly influenced by both the hawthorn species and the analyzed morphological part of the plant (Figure 2).

The content of total polyphenols ranged from 72.0 (flowers *C. laevigata*) to 243.9 (berries *C. laevigata x rhipidophylla x monogyna*) mg GAE/g, flavonoids from 11.9 (leaves *C. rhipidophylla*) to 86.8 (berries *C. x subsphaericea*) mg QE/g, and the total content of procyanidins ranged from 6.5 (flowers *C. laevigata*) to 45.6 (berries *C. rhipidophylla*) mg CYE/g. The highest average content of polyphenols and total proanthocyanidins was found in hawthorn fruits, while the highest content of total flavonoids was found in flowers. In turn, among the species studied, the highest content of total polyphenols was found in fruits and leaves of the fourth hawthorn species (*C. laevigata x rhipidophylla x monogyna*) and flowers of the third species (*C. rhipidophylla*). The highest content of flavonoids was found in fruits of the third species (*C. x subsphaericea*), leaves of the fifth species (*C. x macrocarpa*), and flowers of the fourth species (*C. laevigata x rhipidophylla x monogyna*). In turn, the highest content of total proanthocyanidins was found in fruits of the second species (*C. rhipidophylla*), leaves of the sixth species (*C. laevigata*), and flowers of the third species (*C. x subsphaericea*).

The differences shown may result from genetic variability between different species of the plant or from genetic variability within the same species. This thesis has already been confirmed in studies of hawthorn extracts conducted by other authors. Alirezalu et al. [35], in their studies of leaves collected from several *C. monogyna* trees, found the content of polyphenols and flavonoids ranging from 6.79 to 47.78 mg GAE/g dm and from 4.01 to 7.36 mg QE/g dm, respectively. Similar discrepancies were observed for flowers of the same hawthorn species. A large variation in the content of total polyphenols in leaves and flowers collected from *C. rhipidophylla* trees was shown by Ozyurek et al. [36], who obtained a range of total polyphenol content expressed in Trolox equivalents ranging from 0.29 to 0.56 mmol TE/g (for leaves) and from 0.33 to 0.74 mmol TE/g (for flowers). Environmental conditions, including UV radiation, hydration, temperature, humidity, and access to nutrients available in the soil, can also modify the composition of polyphenolic compounds in hawthorn shrubs and trees. Kirakosyan et al. [37] analyzed the content of total polyphenols and flavonoids in extracts obtained from leaves of *C. laevigata* and *C. monogyna* and noticed an increase in the level of these groups of components under the influence of cold or drought stress. Several studies also report different ranges of polyphenolic compounds depending on the maturity of hawthorn morphological parts at the time of harvest. Luo et al. [38] examined hawthorn fruits, leaves, twigs, and roots collected in the period from May to October and showed significant differences between the harvest dates and the content of total polyphenols and flavonoids, noting the highest levels for fruits, leaves, and twigs in September and for roots in July. The same observations appear in the work of Gao et al. [39]. Important factors influencing the level of polyphenols, flavonoids, and total proanthocyanidins are also the conditions of the storage of raw materials after harvest and their processing techniques. According to Garcia-Mateos et al. [40], the polyphenolic composition of hawthorn morphological parts can be modified by oxidative reactions occurring during processing and storage of the material. Nevertheless, despite the differences shown within the analyzed species and morphological parts, as well as attempts to explain their possible causes, the values obtained in this work are significantly higher compared to the results obtained in the works cited above. Therefore, the implementation of one of the goals of the work, i.e., obtaining high-polyphenolic preparations, was achieved as a result of the use of solid-phase extraction.

### 2.3. Identification of Polyphenolic Compounds

In the previously published works, among all phenolic compounds present in hawthorn, flavonols and proanthocyanidins were considered the main active groups, assigning them the responsibility for the health-promoting effects of this plant [8,10,41]. These categories of compounds in pharmacopoeias are also used for the standardization and quality control of preparations obtained from hawthorn fruits and flowers [9]. The obtained results are summarized in Table 1 and Table S1 and Figures S1–S4.

The UPLC-PDA-MS/MS analysis showed that the quantitative and qualitative profile of identified phenolic compounds was significantly different both among the analyzed morphological parts and selected species. Among the morphological parts, the richest qualitative and quantitative profile was found for preparations obtained from hawthorn fruit, where a total of 18 phenolic compounds were identified, respectively 5 and 5 more compounds than in preparations obtained from leaves and flowers. The most numerous group of phytochemicals in preparations from fruits and leaves were flavan-3-ols and flavonols in terms of quantity. In turn, the main groups of compounds identified in preparations from flowers were flavonols. The highest concentrations of phenolic compounds were also assessed for preparations obtained from fruits. The estimated concentration ranged from 47.74 (*C. laevigata*) to 66.06 (*C. laevigata x rhipidophylla x monogyna*) mg/g and was approximately 1.7 and 1.2 times higher, respectively, compared to the content of polyphenolic compounds in hawthorn leaf and flower preparations. Furthermore, Bekbolatova et al. [10] examined different morphological parts of the hawthorn plant and found the highest concentrations of phenolic compounds in the fruits, for which the values were estimated to be 1.5 and 2.1 times higher, respectively, compared to leaves and flowers. Moreover, Li et al. [41] examined the skin and pulp of hawthorn fruits and found a 5.4-fold higher concentration of polyphenols in the extract from the fruit skin.

Among all phenolic compounds, the highest concentrations in hawthorn fruit preparations were found for 3-*O*-caffeoylquinic acid (32.4% of all identified compounds), quercetin-3-*O*-glucoside (19.5%), and procyanidin dimer (13.9%). The highest concentration of 3-*O*-caffeoylquinic acid was found in *C. laevigata x rhipidophylla x monogyna* fruit (21.39 mg/g). In turn, the phenolic profile studies conducted by Lin et al. [8] and Wen et al. [9] showed a dominant share of proanthocyanidins in *C. pinnatifida* fruits, the concentration of which ranged from 0.10 to 12.40 mg/g, with procyanidin B2 being the highest concentration. In our own studies, the sum of all proanthocyanidins ranged from 9.99 (*C. x subsphaericea*) to 17.41 (*C. rhipidophylla*) mg/g, and the dominant compound was mainly procyanidin dimer (type B). A different view was presented in their analyses by Bekbolatova et al. [10] and Alirezalu et al. [28], who considered the main compound found in hawthorn fruits (*C. almaatensis*, *C. szovitsii*, *C. pentagyna*) to be quercetin 3-*O*-galactoside, the content of which was estimated at the level from 0.38 to 2.94 mg/g.

In the case of hawthorn leaf preparations, the order of the three phenolic compounds found in the highest concentration was as follows: procyanidin dimer (41.3% of all identified compounds) > 4-*O*-caffeolyquinic acid (37.2%) > quercetin-3-*O*-galactoside (4.5%). In turn, the studies conducted by Bekbolatov et al. [10] showed that the dominant polyphenol of hawthorn leaves is quercetin-3-*O*-galactoside. The content of this metabolite in *C. almaatensis* leaves was 2.19 mg/g. In the study by Gao et al. [39], the main compounds found in *C. pinnatifida* leaves included vitexin-2-*O*-rhamnoside, the estimated concentration of which ranged from 4.48 to 5.61 mg/g. This compound was also one of the two dominant compounds in the polyphenolic profile of 14 hawthorn leaf species analyzed by Alirezalu et al. [28]. In our own studies, the presence of this compound was not detected.

Among all phenolic compounds present in the hawthorn flower preparations, the highest concentrations were detected for quercetin-3-*O*-glucoside (31.2% of all identified compounds), 3-*O*-caffeolyquinic acid (21.6%), and 4-*O*-caffeolyquinic acid (18.8%). According to the available literature on the phenolic composition of hawthorn, the main phenolic compound of flowers is quercetin-3-*O*-glucoside [42]. Bekbolatova et al. [10] and Bhri-Sahloul et al. [43] confirmed this thesis for *C. almaatensis* and *C. azarolus* flowers, estimating the content of this compound at a level from 0.25 to 4.12 mg/g. In our own studies, quercetin 3-*O*-glucoside was also one of the dominant compounds and was found in the highest concentration (14.91 mg/g) in *C. laevigata* flowers. On the other hand, different results were obtained by Alirezalu et al. [35] and Froehlicher et al. [44], who studied flowers of 14 hawthorn species and showed that the dominant compound was chlorogenic acid. Its concentration ranged from 0.49 (*C. pseudoheterophylla*) to 12.67 (*C. pseudomelanocarpa*) mg/g. In our own studies, chlorogenic acid derivatives dominated in the obtained preparations.

Considering also the hawthorn species selected in this study, the richest phenolic profile for fruits was demonstrated for preparations obtained from the fourth species (*C. laevigata x rhipidophylla x monogyna*). In the case of preparations from leaves and flowers, for the first hawthorn species—*C. monogyna*. The type of identified phenolic compounds in fruits, leaves, and flowers was basically the same in the six hawthorn species studied, which results from the genetic conditions of the plant. However, great diversity was found in the content and ratio of specific phytochemicals. Significant differences in the amount of polyphenolic compounds in different hawthorn species were also observed in many other works [18,45,46]. As mentioned earlier, the concentration of polyphenols depends on the place of cultivation, the stage of maturity, the storage conditions of the raw materials, and the method of preparing the extract. These factors could therefore have a significant impact on the differences in the content of individual groups of polyphenolic compounds among the six analyzed hawthorn species. Moreover, as can be seen, significantly higher concentrations of phenolic compounds were noted in this work compared to the cited works. This is mainly due to the use of the SPE method using the Amberlite XAD-2 bed, which allowed for the removal of ballast substances such as proteins or fats, leading to an increase in the concentration of phenolic compounds in terms of the dry mass of the preparation. This makes the obtained preparations a basis for the design of nutraceuticals, where a high dose of phenolic compounds is obtained at a low concentration of the preparation.

In addition, it can be noted that in the obtained preparations, in comparison to the cited works, mainly phenolic acids dominated. This can be explained by the type of sorbent used to purify phenolic fractions. Previous works have shown a higher ability to absorb compounds, mainly phenolic acids, by polymeric sorbents such as the Amberlite XAD-2 used in this work compared to other deposits, such as C18, which was attributed to their aromatic structure, which can sorb aromatic phenolic compounds via π-π interactions [47].

### 2.4. Antioxidant Activity

Preparations obtained from hawthorn fruits, leaves, and flowers were subjected to antioxidant activity assessment using five methods (Table 2). Analyzing the results of our own research, it can be stated that among the morphological parts tested, the highest antioxidant activity was characteristic of preparations from hawthorn fruits, while among the species, the highest potential was demonstrated for *C. laevigata x rhipidophylla x monogyna* (for fruits and leaves) and *C. subsphaericea* (for flowers).

On the other hand, when analyzing individual methods, the highest activity in the ABTS and CUPRAC methods was characteristic of hawthorn fruit preparations, with an average value of 7.25 and 6.38 mmol TE/g, respectively. For both methods, the highest activity was characteristic of preparations from the species *C. laevigata x rhipidophylla x monogyna*. In turn, the lowest (by about 1.9 times compared to fruits) values were shown for hawthorn flower preparations. The obtained results are much higher in comparison to previous reports. Luo et al. [38] showed values of 25.9 and 16.5 times lower for extracts from *C. pinnatifida* fruits and leaves for the ABTS method. Also, for the CUPRAC method, the values were much lower in other works. Ozyurek et al. [36], analyzing the leaves and flowers of 17 hawthorn species, obtained values of 14.2 and 10.5 times lower, respectively.

For the next method of iron ion chelation, the highest activity expressed as IC_50_ values was also shown for fruits, with an average value of 678.93 µg/mL, including the species *C. laevigata x rhipidophylla x monogyna*. To date, only the ability to chelate iron ions has been analyzed for leaves. Kallassy et al. [14] obtained IC_50_ values ranging from 500.0 to 1500.0 µg/mL for methanol and ethanol extracts of *C. azarolus* leaves. For the last two methods, O_2_˙^−^ and OH˙ radical scavenging, the highest potential was also shown for fruit preparations, with average values of 381.05 and 116.02 µg/mL, respectively. In both methods, the highest potential was shown for the species *C. rihipidophylla*. For both the O_2_˙^−^ and OH˙ radical scavenging method and iron ion chelation, the lowest activity was demonstrated for hawthorn flower preparations. The mean values were 1.2, 1.3, and 3.2 times lower, respectively. The ability to scavenge O_2_˙^−^ anion radicals and OH˙ radicals by hawthorn leaf and flower extracts has not been assessed by other authors so far. In turn, comparable results for fruits were presented in the work of Lin et al. [8], where for the O_2_˙^−^ and OH˙ radical scavenging test, the values for ethanol extracts from *C. pinnatifida* fruits were >400 and 139.03 µg/mL, respectively. In turn, high O_2_˙^−^ and OH˙ radical scavenging capacity was presented by Liu et al. [48] for the procyanidin fraction from *C. pinnatifida* fruits. The values obtained by them ranged from 23.0 to 110.0 µg/mL for the O_2_˙^−^ test and from 14.0 to 117.0 µg/mL for the OH˙ test, respectively.

Many studies have shown that hawthorn has a strong antioxidant effect, which is mainly shaped by the content and profile of individual polyphenol groups. Our own studies have shown that the antioxidant potential of hawthorn fruit, leaf, and flower preparations mainly increases with the increase in flavan-3-ol content. Mizuno et al. [49] and Wu et al. [50] found that procyanidin polymers have high antioxidant activity. In the available literature, there have been reports of a strong correlation between the high content of proanthocyanidins and the antiradical properties of, among others, red grapes [51], rosehips [52], and chokeberries [53]. Similar observations concern the assessment of the phenolic profile and antioxidant activity of hawthorn. In the work of Bahri-Sahloul et al. [43], a strong positive correlation was found between antioxidant activity and the total content of proanthocyanidins, isoquercetin, and procyanidin B2 identified in *C. monogyna* flowers. Bardakci et al. [54] also found that the antioxidant activity of fruit extracts was strongly correlated with the content of procyanidin B2. Among the procyanidins identified in fruits, leaves, and flowers, Froehlicher et al. [44] found that (−)-epicatechin is the most effective antioxidant compound of hawthorn. Apart from polyphenolic compounds, antioxidant activity also depends on genetic and species factors, climatic conditions, and the concentration of other secondary metabolites such as vitamin C and carotenoids [28].

The above results of the evaluation of antioxidant activity of preparations from hawthorn fruit, leaves, and flowers prove that the selected extraction method allows for a significant increase in the antiradical potential of plant-derived powders. Thanks to this, the obtained preparations could be successfully used as a natural food additive inhibiting oxidative changes and thus extending the durability of the product, as well as an additive to functional food dedicated to people suffering from diseases resulting from the disturbance of internal redox homeostasis.

### 2.5. Anti-Diabetic and Anti-Inflammatory Activity

One of the important therapeutic strategies aimed at reducing the incidence of type II diabetes and inflammation is the use of plant compounds to inhibit the activity of enzymes involved in the pathogenesis of these diseases. Hence, the potential of the obtained hawthorn preparations as inhibitors of α-amylase, α-glucosidase, and trypsin was assessed in the studies (Table 2). The highest activity of inhibiting α-amylase and α-glucosidase was characterized by preparations from fruits. The obtained IC_50_ values for this group of samples ranged from 0.48 to 1.11 mg/mL for inhibiting α-amylase activity and from 0.87 to 1.57 mg/mL in inhibiting α-glucosidase activity. In both assays, the highest activity was observed in the fruits of *C. laevigata x rhipidophylla x monogyna*, and the lowest in *C. laevigata*. The mean values for leaves and flowers were 1.14 and 1.07 mg/mL (for α-amylase) and 1.86 and 2.02 mg/mL (for α-glucosidase), respectively.

**Figure 3:**
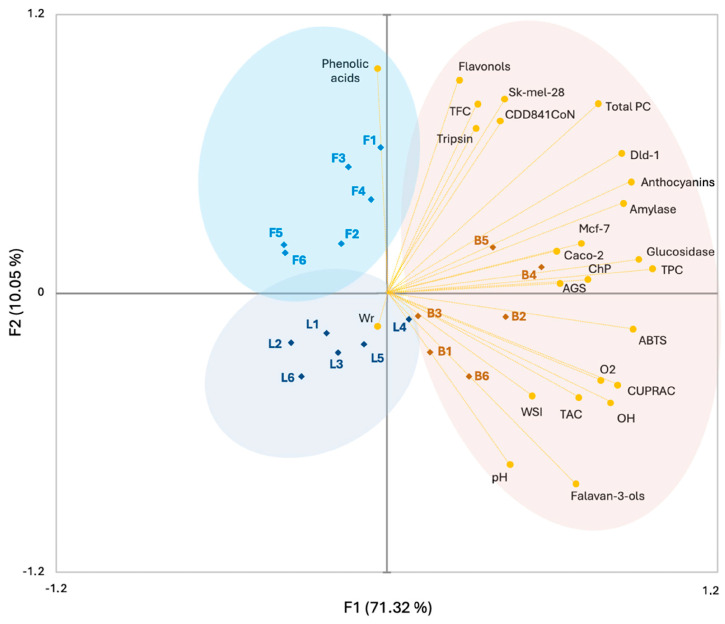
PCA analysis between studied hawthorn species, morphological parts, and analyzed parameters. Explanations: B, Berries; L, Leaves; F, Flowers; 1–6, hawthorn species (see Section 2.2. Plant material).

**Figure 4:**
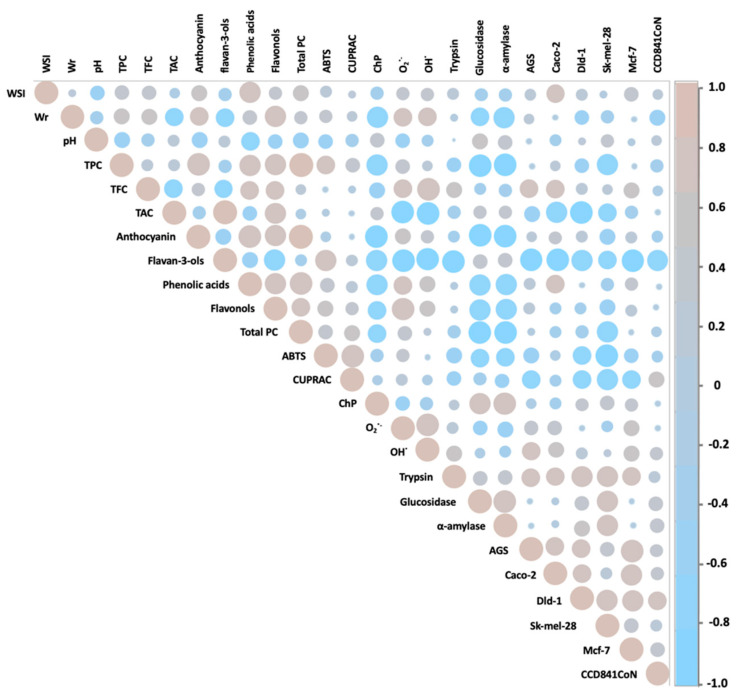
Pearson correlation illustrating the relationships between the studied variables for preparations obtained from hawthorn berries.

**Figure 5:**
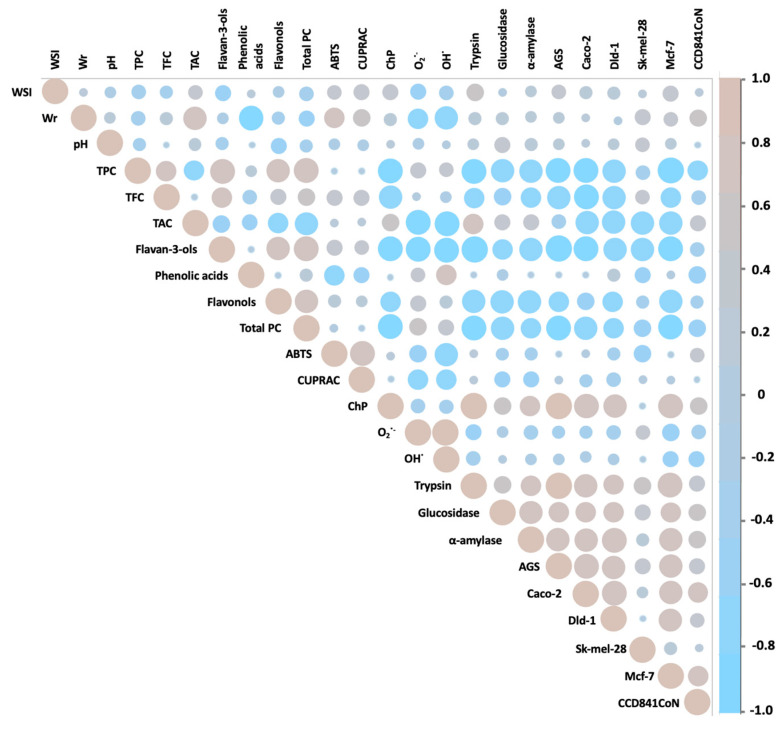
Pearson correlation illustrating the relationships between the studied variables for preparations obtained from hawthorn leaves.

**Figure 6:**
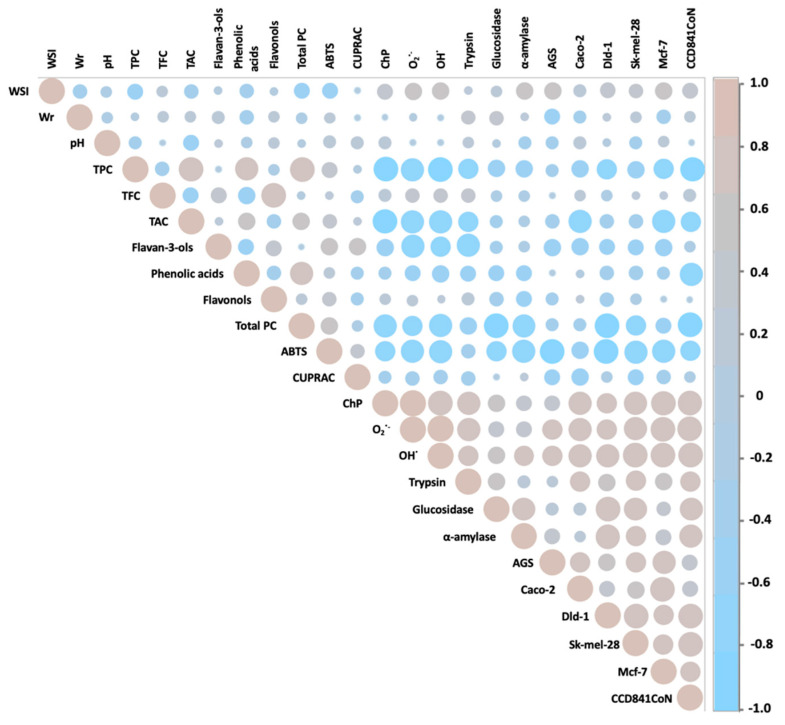
Pearson correlation illustrating the relationships between the studied variables for preparations obtained from hawthorn flowers.

The antidiabetic potential of hawthorn has only been appreciated in recent years. However, only the fruits and flowers of *C. pinnatifida*, *C. monogyna*, and *C. laciciata* species have been analyzed so far. The IC_50_ values of glucosidase inhibition by the abovementioned hawthorn species were 0.12–0.34 mg/mL for fruits [8,55] and 4.6 mg/mL for hawthorn flowers [56], respectively. In turn, in the case of α-amylase activity inhibition, concentrations of 0.44 mg/mL for fruits [57] and 0.52 mg/mL for flowers [56] were obtained. The abovementioned values from previously published works are comparable to those obtained in this work. At the same time, Lin et al. [8] proved that the antidiabetic activity of hawthorn fruits depends on the extraction method and the polyphenolic composition of the extracts obtained. This was also confirmed by Paun et al. [57], who showed that the antidiabetic activity of the hawthorn fruit extracts tested depended mainly on the concentration of phenolic acids and the formation of hydrogen bonds between amino acid residues in the active site of the enzymes and the -OH groups of phenolic compounds. In our studies, we showed that the activity against both enzymes significantly depended on the content of anthocyanins and flavonols in the case of fruit preparations, on the content of flavan-3-ols and flavonols in the case of leaf preparations, and on the presence of flavonols in hawthorn flower preparations (Figure 3, Figure 4, Figure 5 and Figure 6). For the above groups of compounds present in the tested preparations, a hypoglycemic effect had already been demonstrated [58,59].

The assessment of anti-inflammatory activity by measuring the ability of preparations to inhibit trypsin activity was assessed for the first time for hawthorn. Unlike in the case of hypoglycemic activity, the highest anti-inflammatory activity was demonstrated for the preparation from hawthorn flowers, *C. monogyna*, obtaining an IC_50_ value of 0.69 mg/mL, with the range for the entire group of preparations from flowers equal to 0.69–4.95 mg/mL. On the other hand, the average values for preparations from hawthorn fruits and leaves were comparable and amounted to 2.26 mg/mL. Previously, Amrati et al. [60] and Wyspiańska et al. [61] demonstrated a strong anti-inflammatory potential of extracts from the leaves, bark, and stems of hawthorn *C. monogyna* by inhibiting COX-1 and COX-2, key enzymes involved in the inflammatory process. However, the cited studies did not demonstrate anti-inflammatory abilities for hawthorn fruits. In both studies, the demonstrated activity was attributed to the presence of proanthocyanidins, in particular procyanidin B2. In our studies, we also noticed a strong effect of flavan-3-ols on trypsin activity (Figure 4, Figure 5 and Figure 6). Several studies on the anti-inflammatory activity of isolated proanthocyanidins have already been conducted, and it has been proven that the activity of this group of compounds is mainly based on the inhibition of NF-κB and MAPK pathways, blocking the expression of mRNA of pro-inflammatory cytokines and inflammatory products of prostaglandins COX-2 [62].

### 2.6. Anticancer Activity

A significant challenge of modern science is the search for new sources of phytochemicals that can become the basis for the development of chemopreventive and chemotherapeutic procedures. Therefore, preparations from the fruits, leaves, and flowers of six hawthorn species were used to assess the ability to inhibit cancer cell viability (Table 3).

Among the five cancer cell lines, the highest sensitivity to the tested preparations was characteristic of the colon cancer cell line (Caco-2). The highest average value of cell viability inhibition after treatment with preparations from fruits (175.43 μg/mL) and flowers (187.83 μg/mL) was noted for the selected line. The highest potential among the analyzed fruits was characteristic of the fruits of species 2 (*C. rhipidophylla*) and flowers of species 4 (*C. laevigata x rhipidophylla x monogyna*). In the case of hawthorn leaves, the highest sensitivity was characteristic of the AGS line (with an average value of 224.87 μg/mL), especially the fourth hawthorn species (*C. laevigata x rhipidophylla x monogyna*). In turn, the lowest potential for inhibiting cell viability after treatment with the tested preparations was demonstrated for the SK-mel-28 line. The average IC_50_ values for fruit, leaf, and flower preparations were 368.28, 407.12, and 349.55 μg/mL, respectively. The Sk-mel-28 line belongs to the group of malignant tumors with a high rate of resistance to chemotherapy; hence the lowest sensitivity of the tested line to hawthorn preparations can be explained.

As for the safety of the developed preparations, by assessing their ability to inhibit the viability of healthy colon epithelial cells (CCD841CoN line), the IC_50_ values were in a comparable range (from 435.47 to >500 μg/mL) for preparations from fruits, leaves, and flowers. Importantly, the obtained results in most cases were higher compared to the cytotoxic concentration estimated for cancer cell lines, which indicates high selectivity of the preparations for human cancer cells.

From the literature review, it can be seen that, to date, the cytotoxicity of hawthorn extracts has been tested against seven cancer cell lines, such as colon cancer cells (HCT-116, HT-29), prostate (PC3), lung (PC14), breast (MCF-7), ovarian (SKOV-3), and T lymphocyte leukemia (Jurkat). High antiproliferative potential was found for *C. azarolus* leaf extracts. The estimated IC_50_ values ranged from 13 to 200 µg/mL against Jurkat cells, from 30 to 60 µg/mL against HCT-116 cells, and from 45 to 65 µg/mL against HT-29 cells [14,63]. In turn, in studies of the activity of the polyphenolic fraction from the peel of *C. pinnatifida* fruit against the MCF-7 and SKOV-3 cell lines, IC_50_ values of 2.76 and 80.11 μg/mL were obtained, respectively [41,64]. The abovementioned concentrations indicate a high anticancer potential of hawthorn fruits, leaves, and flowers, comparable to and higher in comparison with the results presented in this paper. This diverse effect of the extracts on the tested cell lines is most likely related to the different classes of polyphenolic components present in the morphological parts of the hawthorn species studied, as well as to differences in the methodology for assessing the viability of cancer cells. In order to fully understand the anticancer activity of the tested preparations, it would also be necessary to estimate the mechanism of their activity in the future. In our previous studies of activity against glioblastoma cells (line U87MG), we showed that polyphenolic preparations from hawthorn fruit have the ability to promote the cleavage of poly(ADP-ribose) polymerase 1 (PARP1) associated with apoptosis and to inhibit the activity of pro-survival kinases, focal adhesion kinase (FAK), and protein kinase B (PKB), which indicates the suppression of the proliferative and invasive potential of the tested glioblastoma multiforme cells; this effect was associated with the concentration of proanthocyanidins in the preparations [7]. In this work, the correlation analysis (Figure 4, Figure 5 and Figure 6) showed that the cytotoxic activity towards the tested cancer cell lines correlated mainly with the content of flavan-3-ols for fruits, with the content of flavan-3-ols, flavonols, and total PC for leaves, and with the content of total PC for flowers. Previously published reports by other groups have shown that flavan-3-ols inhibit cell viability and/or induce apoptosis in breast cancer cells (MCF-7, MDA-MB-468), lung cancer (A427), prostate cancer (DU145), colon cancer (HCT-8, HT29, Caco-2), and bladder cancer (BIU87) [65]. It has been shown that the chemical structure of polyphenols determines their anticancer efficacy, and in the case of oligomeric flavan-3-ols, their degree of polymerization positively correlates with reduced viability of cancer cells [66]. In our previous studies on the polyphenolic composition of hawthorn seeds, we also found a significant relationship between anticancer activity and the content of flavan-3-ols, mainly procyanidin dimer type-B [67]. The presented data may serve in the future, after further analysis, as a basis for the development of a potential anticancer therapeutic strategy based on a single compound or a combination of several compounds isolated from hawthorn fruits, leaves, or flowers.

### 2.7. PCA

Principal component analysis (PCA) was used to analyze similarities and differences between the studied parameters and species (Figure 3). Using the PCA graph, two component areas, PC1 (71.32%) and PC2 (10.05%), were distinguished, accounting for 81.37% of the total data variance. Moreover, the existence of three clusters was demonstrated, the composition of which significantly depended on the analyzed morphological part of the hawthorn bush. The first distinguished group consisted of preparations obtained from the fruits of all hawthorn species. These preparations were characterized by a high content of most of the assessed groups of phenolic compounds (anthocyanins, flavan-3-ols, flavonoles, TPC, TFC, TAC), as well as total polyphenol content (Total PC), which correlated with most of the assessed parameters, such as physicochemical (pH, WSI), antioxidant activity (ABTS, CUPRAC, ChP, O_2_˙^−^, OH˙), antidiabetic (α-amylase, α-glucoamylase), anti-inflammatory (trypsin), and anticancer (AGS, Caco-2, Ht-29, Mcf-7, Sk-mel-28), including high activity against healthy cells (CCD841CoN). The next two groups were composed of preparations obtained from hawthorn leaves and flowers, respectively. Additionally, the group of preparations from leaves correlated with a high value of the Wr parameter. In turn, the group created from preparations from flowers was characterized by a high value of phenolic acid content. Overall, the presented biplot confirmed our earlier conclusions regarding the relationship between the examined morphological part of hawthorn bushes and the content of bioactive compounds and health-promoting properties. It can also be concluded that it is hawthorn fruit preparations, apart from having the richest polyphenol profile, that mainly show the highest health-promoting potential.

## 3. Materials and Methods

### 3.1. Materials and Reagents

Quercetin, gallic acid, cyanidin chloride, neocuproine, ferrozine, NBT (nitrotetrazolium blue chloride), PMS (phenazine methosulfate), NADH (*β*-Nicotinamide adenine dinucleotide, reduced disodium salt hydrate), 2-Deoxy-D-ribose, and EDTA (ethylenediaminetetraacetic acid disodium salt dihydrate), Dulbecco’s Modified Eagle Medium-GlutaMAX-1 (DMEM), RPMI 1640 Media, 0.25% trypsin-EDTA, fetal bovine serum (FBS), phosphate-buffered saline (PBS), and antibiotics (100 U/mL) were purchased from Sigma-Aldrich (Steinheim, Germany). CellTiter 96^®^ AQueous Non-Radioactive Cell Proliferation Assay was purchased from Promega (Madison, WI, USA). All other chemicals were purchased from Chempur (Piekary Śląskie, Poland).

### 3.2. Plant Material

The study used material from morphological parts, such as berries, leaves, and flowers from six species of hawthorn (*Crataegus*): (1) *C. monogyna*, (2) *C. rhipidophylla*, (3) *C. x subsphaericea,* (4) *C. laevigata x rhipidophylla x monogyna*, (5) *C. macrocarpa*, and (6) *C. laevigata*. Samples were collected from bushes located near Rzeszów (Poland) in 2016. After being delivered to the laboratory, the collected material was freeze-dried (ALPHA 1–2 LD plus, Martin Christ, Germany) and ground (seeds were removed from the fruits before grinding).

### 3.3. Preparation of Extract

Polyphenolic preparation from hawthorn fruits, leaves, and flowers was obtained by solid-phase extraction (SPE) according to our previous reports [67,68]. Briefly, 30 g of ground material was combined with 150 mL of 50% methanol and sonicated (ultrasonic bath, Sonic 10, Polsonic, Poland) for 20 min at 30 °C. The suspension was centrifuged (Centrifuge 5430, Eppendorf, Hamburg, Germany), the supernatant was collected, and the residue was resubjected to the above extraction using 150 mL of 70% methanol. After combining the centrifuged extracts, the methanol was evaporated in a rotary evaporator (R-215 Rotavapor System, Buchi, Switzerland) at 40 °C. Concentrated samples were applied to a glass column (50 mm, 500 mm; Sigma-Aldrich, Steinheim, Germany) filled with a bed of Amberlite XAD-2 (pore size 90 Å, particle size 0.3–1.2 mm), which had been previously conditioned with methanol and equilibrated with water. The polyphenolic fraction was eluted with methanol. For hawthorn fruit extracts, both water and methanol were acidified by adding formic acid (1%). In order to obtain powdered preparations, the collected polyphenolic fractions were lyophilized.

### 3.4. Measurement of Physicochemical Parameters

#### 3.4.1. Determination of pH Value

The pH value was determined by the potentiometric method using a pH meter (CPC-411, Elmetron, Poland).

#### 3.4.2. Determination of the Water Solubility Index

The determination of the water solubility index (WSI) was performed using the centrifuge method. A total of 10 mL of water was added to 1 g of the polyphenol preparation and then mixed for 10 min. After this time, the samples were centrifuged at 3000 rpm for 10 min, and the obtained supernatant was dried in a laboratory dryer (ED 115, Binder, Tuttlingen, Germany) to a constant mass. The water solubility index was calculated based on the dry mass of the sample and the mass of the dried supernatant.

#### 3.4.3. Determination of Equilibrium Moisture Content

The equilibrium moisture content (Wr) was determined using a moisture analyzer (Sartorius MA35, Kostrzyn, Poland). The moisture content was calculated based on the difference in weight before and after drying.

### 3.5. Determination of Polyphenol Groups

#### 3.5.1. Determination of Total Phenolic Content

The measurement was performed according to the method of Gao et al. [69]. In brief, polyphenolic preparations were mixed with distilled water, Folin-Ciocalteau reagent, and sodium carbonate. After 1 h, the absorbance was measured at a wavelength of 765 nm using a UV–VIS spectrometer (Type UV2900, Hitachi, Tokyo, Japan). The calibration curve parameters were as follows: y = 0.0330x + 0.06, R^2^ = 0.998. The results were expressed in mg gallic acid (GAE)/g dm.

#### 3.5.2. Determination of Total Flavonoid Content

The measurement was performed according to the method of Chang et al. [70]. In brief, polyphenolic preparations were mixed with ethanol, aluminum chloride, distilled water, and sodium acetate. After 30 min, the absorbance was measured at 415 nm. The calibration curve parameters were as follows: y = 0.0060x + 0.0120, R^2^ = 0.997. The results were expressed in mg quercetin (QE)/g dm.

#### 3.5.3. Determination of Proanthocyanidin Content

The measurement was performed according to the method of Chang et al. [70]. In brief, polyphenolic preparations were mixed with BuOH, iron (III) ammonium sulfate, and distilled water. After 45 min, the absorbance was measured at 550 nm. The calibration curve parameters were as follows: y = 0.0054x + 0.0426, R^2^ = 0.996. The results were expressed in mg cyanidin chloride (CYE)/g dm.

### 3.6. Determination of Phenols Profile by UPLC-Q-TOF-MS

Polyphenolic compounds were analyzed by ultra-performance liquid chromatography coupled to mass spectrometry using an ACQUITY device (Waters, Milford, MA, USA) according to the work of Żurek et al. [67]. The chromatograph was equipped with a binary pump manager, a column manager, a sample manager, a photodiode array detector (PDA), and a tandem quadrupole mass spectrometer in the form of a double quadrupole (TQD) with an electrospray ionization (ESI) source operating in positive and negative ion sweep mode. Phenolic compounds were separated using a BEH RP C18 UPLC column (Waters, Milford, USA). For the analysis of anthocyanins, the mobile phase consisted of 2% formic acid in water (solvent A) and 40% acetonitrile in water with 2% formic acid (solvent B). For other polyphenolic compounds, the mobile phase was 0.1% formic acid in water (solvent A) and 40% acetonitrile in water with 0.1% formic acid (solvent B). The flow rate of the mobile phase was kept constant at 0.35 mL/min throughout the analysis (9.5 min). The sample injection volume was 5 µL. The column temperature was maintained at 50 °C. The obtained data were processed in Waters MassLynx v.4.1 (Waters Corporation, MA, USA). Results are expressed as mg/g dm.

### 3.7. Determination of Antioxidant Activity

The measurement of ABTS^•+^ radical scavenging activity was performed by mixing the polyphenol preparations with ABTS^•+^ solution. After 6 min, the absorbance at 734 nm was measured. The results were expressed as mmol Trolox (TE)/g dm. [71]. The measurement of copper ion reduction (CUPRAC) was performed by mixing the polyphenol preparations with neocuproine solution, copper chloride, and acetate buffer. After 30 min, the absorbance was measured at 450 nm. The results were expressed as mmol TE/g dm [72]. The measurement of the chelating ability of ferrous ion was performed by mixing the polyphenol preparations with iron II sulfate and ferrozine solution. After 10 min, the absorbance was measured at 562 nm. The results were expressed as the IC_50_ [73]. The measurement of the superoxide (O_2_˙^−^) radical scavenging activity assay was performed by mixing the polyphenol preparations with nitrotetrazolium blue chloride, β-Nicotinamide adenine dinucleotide sodium salt, and phenazine methosulfate. After 5 min, the absorbance was measured at 560 nm. The results were expressed as the IC_50_ [74]. The measurement of the hydroxyl (OH˙) radical scavenging activity assay was performed by mixing the polyphenol preparations with 2-deoxyribose, iron ammonium sulphate, EDTA tetrasodium salt, ascorbic acid, perhydrol, trichloroacetic acid, and thiobarbituric acid. After 30 min, the absorbance was measured at 532 nm. The results were expressed as IC_50_.

### 3.8. Measurement of Health-Promoting Activity

The inhibition of α-amylase activity was measured by mixing the polyphenol preparations with porcine pancreatic α-amylase (10 μg/mL), starch solution, ethanol, and distilled water. After 25 min, the absorbance was measured at 510 nm. The inhibition of α-glucosidase activity was measured by mixing the polyphenol preparations with α-glucosidases (10 μg/mL) and 4-Nitrophenyl β-D-glucopyranoside. After 20 min, the absorbance was measured at 405 nm. Trypsin activity inhibition was measured by mixing polyphenol preparations with bovine pancreatic α-trypsin (10 μg/mL) and N-α-benzoyl-L-arginine 4-nitroanilide hydrochloride. After 10 min, absorbance was measured at 410 nm. All results were expressed as IC_50_.

### 3.9. Measurement of Cytotoxic Activity Against Human Cells

Six cell lines were selected for the study: breast cancer cell line (Mcf-7), gastric cancer (AGS), two colorectal cancer cell lines (Caco-2, Dld-1), melanoma (Sk-mel-28), and one line of healthy colonic epithelial cells (CCD841CoN). Cell lines were cultured in DMEM, McCoy’s, and RPMI media supplemented with fetal bovine serum and penicillin/streptomycin. Cell growth was ensured by providing appropriate conditions (37 °C, 5% CO_2_, 95% humidity) in an incubator (CB170, Binder, Germany).

To assess cell viability, selected cell lines were seeded into 96-well plates (8 × 10^4^ cells/well) and allowed to adhere in the appropriate medium under controlled conditions for 24 h. After this time, the culture medium was replaced with polyphenol preparations dissolved in the medium and left in contact with the cells for 24 h. Then, the extracts were removed, the cells were washed with phosphate buffer, and fresh medium without phenol red in which the MTS reagent (Promega) was dissolved was added. After 1 h, absorbance at 490 nm was measured using a microplate reader (SmartReader 96 Microplate Absorbance Reader, Edison, NJ, USA). Cells without extract treatment were used as a control. The results were expressed as IC_50_.

### 3.10. Statistical Analysis

All determinations were performed in at least three replicates, presenting the results as means and standard deviation (SD). Statistical significance was analyzed using Duncan’s test, correlation using the Pearson correlation matrix, and principal component analysis (PCA). All tests were performed in Statistica v. 13.3 (StatSoft, Kraków, Poland).

## 4. Conclusions

This work provides valuable and still missing data on the possibilities of using different morphological parts of hawthorn bushes, i.e., berries, leaves, and flowers, to design highly bioactive nutraceuticals. Using solid-phase extraction, preparations with a concentrated content of polyphenolic compounds were obtained per mass of the preparation. A total of 27 phenolic compounds were identified in the obtained preparations, where preparations obtained from hawthorn fruits, especially the species *C. laevigata x rhipidophylla x monogyna*, stood out in terms of quantity and quality. In the assessment of health-promoting activity, all preparations were characterized by high antioxidant activity; they also showed the ability to inhibit the activity of α-glucosidase and α-amylase, but lower in relation to trypsin activity. In turn, among the tested cancer lines, the highest activity was demonstrated against colon cancer cells (Caco-2) and stomach (AGS), at the same time proving high biocompatibility with normal human colon cells. The proven properties were strongly dependent on the analyzed morphological part and hawthorn species, with the highest properties being characteristic of hawthorn fruit preparations, mainly the species *C. laevigata x rhipidophylla x monogyna*, which was confirmed by the PCA principal component analysis. Physicochemical parameters of the preparations, such as water solubility index, pH, and equilibrium moisture, were similar within the analyzed morphological parts and species and were characterized by high functional properties.

The demonstrated phenolic composition, health-promoting activity, and physicochemical properties of hawthorn preparations may constitute the basis for their commercial use in the food, pharmaceutical, and cosmetic industries. The final preparations obtained may constitute a more attractive technological and health substrate for the development of nutraceuticals, dietary supplements, and hygiene products with targeted health-promoting effects.

## Figures and Tables

**Table 1:** Detailed quantification of phenolic compounds content (mg/g d.m.) in preparations from berries, leaves, and flowers of six hawthorn species.

n			Species of Hawthorn					
			*C. monogyna*	*C. rhipidophylla*	*C. x subsphaericea*	*C. laevigata x rhipidophylla x monogyna*	*C. x macrocarpa*	*C. laevigata*
Berries								
*Anthocyanins*	1 *	C3Glu	0.56 ± 0.01 c	0.53 ± 0.00 b	0.53 ± 0.00 bc	0.74 ± 0.00 e	0.66 ± 0.01 d	0.38 ± 0.00 a
	2	P3R	0.02 ± 0.00 b	0.02 ± 0.00 a	0.02 ± 0.00 a	0.02 ± 0.00 c	0.02 ± 0.00 a	0.02 ± 0.00 a
	3	C3A	0.03 ± 0.00 b	0.03 ± 0.0 b	0.03 ± 0.00 a	0.03 ± 0.00 c	0.03 ± 0.00 a	0.03 ± 0.00 c
	4	P3Glu	0.05 ± 0.00 e	0.05 ± 0.00 d	0.04 ± 0.00 c	0.04 ± 0.00 b	0.03 ± 0.00 a	0.03 ± 0.00 a
		Sum	0.66 ± 0.01 c	0.63 ± 0.00 b	0.63 ± 0.00 b	0.84 ± 0.00 e	0.74 ± 0.01 d	0.46 ± 0.00 a
*Flavan-3-ols*	5	PT	4.42 ± 0.02 b	7.40 ± 0.00 c	4.18 ± 0.00 b	1.28 ± 0.01 a	10.73 ± 0.00 d	13.18 ± 0.00 e
	6	PD	6.76 ± 0.03 d	10.01 ± 0.03 f	5.81 ± 0.01 c	9.20 ± 0.01 e	1.51 ± 0.02 a	2.66 ± 0.04 b
	7	C(+)	8.31 ± 0.01 b	10.35 ± 0.03 d	5.97 ± 0.01 a	8.58 ± 0.03 b	9.97 ± 0.00 c	10.21 ± 0.04 d
	10	C	0.56 ± 0.00 e	0.14 ± 0.02 c	0.04 ± 0.00 a	0.05 ± 0.00 a	0.30 ± 0.01 d	0.06 ± 0.00 b
	11	4EGGlu	0.50 ± 0.01 a	1.28 ± 0.02 e	0.91 ± 0.03 c	1.43 ± 0.00 f	1.06 ± 0.01 d	0.65 ± 0.00 b
		Sum	20.55 ± 0.08 b	29.18 ± 0.10 e	16.91 ± 0.05 a	20.55 ± 0.05 b	23.70 ± 0.03 c	26.76 ± 0.08 d
*Phenolic acids*	12	QA	0.22 ± 0.04 e	0.11 ± 0.01 bc	0.09 ± 0.01 b	0.09 ± 0.00 a	0.11 ± 0.01 c	0.13 ± 0.01 d
	13	3PA	0.30 ± 0.00 a	0.42 ± 0.01 b	0.56 ± 0.01 d	0.30 ± 0.01 a	0.54 ± 0.00 d	0.50 ± 0.01 c
	14	4CA	6.52 ± 0.07 d	4.96 ± 0.00 b	5.77 ± 0.00 c	3.72 ± 0.07 a	12.32 ± 0.02 e	6.83 ± 0.01 d
	15	3CA	9.68 ± 0.02 c	10.42 ± 0.06 d	14.96 ± 0.01 e	21.39 ± 0.04 f	6.84 ± 0.02 b	5.42 ± 0.00 a
		Sum	16.73 ± 0.13 b	15.91 ± 0.09 b	21.38 ± 0.03 d	25.50 ± 0.13 e	19.80 ± 0.05 c	12.88 ± 0.03 a
*Flavonols*	17	L8Glu	3.70 ± 0.05 c	0.49 ± 0.01 a	3.60 ± 0.02 d	5.66 ± 0.01 e	5.73 ± 0.07 e	0.56 ± 0.03 b
	19	N7Glu	0.39 ± 0.02 c	0.13 ± 0.00 b	0.09 ± 0.03 a	0.14 ± 0.01 b	0.69 ± 0.03 d	0.09 ± 0.01 a
	21	Q3Glu	8.04 ± 0.01 c	4.09 ± 0.02 a	8.47 ± 0.02 c	12.90 ± 0.03 d	14.28 ± 0.01 e	6.14 ± 0.03 b
	23	A7R	0.21 ± 0.00 b	1.42 ± 0.02 e	0.39 ± 0.03 c	0.06 ± 0.00 a	0.34 ± 0.00 c	0.75 ± 0.01 d
	24	A8Glu	0.37 ± 0.09 c	0.38 ± 0.00 c	0.14 ± 0.01 b	0.43 ± 0.01 d	0.17 ± 0.03 b	0.11 ± 0.01 a
		Sum	12.71 ± 0.17 c	6.51 ± 0.05 a	12.70 ± 0.10 c	19.18 ± 0.05 d	21.21 ± 0.14 e	7.65 ± 0.09 b
		PC (mg/g dm)	50.64 ± 0.38 b	52.22 ± 0.23 d	51.61 ± 0.18 c	66.06 ± 0.23 f	55.45 ± 0.23 e	47.74 ± 0.20 a
Leaves								
*Flavan-3-ols*	5	PT	0.66 ± 0.01 c	0.48 ± 0.00 a	0.75 ± 0.05 d	0.81 ± 0.01 e	0.77 ± 0.01 d	0.51 ± 0.01 b
	6	PD	5.66 ± 0.01 b	6.11 ± 0.05 b	52.33 ± 0.02 a	16.27 ± 0.01 d	5.09 ± 0.00 a	12.87 ± 0.04 c
	8	E(−)	1.10 ± 0.07 d	0.89 ± 0.00 b	0.98 ± 0.03 c	1.12 ± 0.00 d	0.98 ± 0.03 c	0.80 ± 0.00 a
	9	Ptet	2.43 ± 0.45 d	2.64 ± 0.02 e	2.03 ± 0.27 c	1.46± 0.00 b	2.20 ± 0.31 d	0.68 ± 0.10 a
	11	4EGGlu	9.16 ± 0.06 c	8.40 ± 0.01 b	9.91 ± 0.03 c	0.19 ± 0.01 a	11.43 ± 0.00 d	0.19 ± 0.00 a
		Sum	19.01 ± 0.61 bc	18.51 ± 0.09 b	18.90 ± 0.40 b	20.86 ± 0.03 d	20.48 ± 0.35 c	15.05 ± 0.16 a
*Phenolic acids*	13	3PA	2.33 ± 0.14 e	1.86 ± 0.01 d	1.45 ± 0.00 c	1.02 ± 0.01 a	1.02 ± 0.02 a	1.28 ± 0.01 b
	14	4CA	13.43 ± 0.02 c	13.49 ± 0.41 c	13.53 ± 0.01 c	14.66 ±0.12 d	12.27 ± 0.02 a	12.91 ± 0.05 b
	15	3CA	0.40 ± 0.02 c	0.12 ± 0.00 a	0.10 ± 0.12 a	0.30 ± 0.00 b	0.50 ± 0.00 d	0.28 ± 0.01 b
	16	3,4CA	1.15 ± 0.15 d	0.83 ± 0.02 c	0.74 ± 0.00 b	0.43 ± 0.00 a	0.73 ± 0.06 b	1.37 ± 0.01 e
		Sum	17.31 ± 0.32 d	0.16 ± 0.04 c	15.82 ± 0.15 b	16.41 ± 0.14 c	14.51 ± 0.10 a	15.83 ± 0.09 b
*Flavonols*	18	L7Glu	0.20 ± 0.02 b	0.52 ± 0.00 c	0.07 ± 0.00 a	0.05 ± 0.00 a	0.21 ± 0.01 b	nd
	21	Q3Glu	0.17 ± 0.00 a	0.41 ± 0.03 d	0.24 ± 0.01 b	0.33 ± 0.03 c	0.21 ± 0.02 b	0.45 ± 0.02 d
	22	Q3G	0.68 ± 0.02 a	0.70 ± 0.00 b	0.70 ± 0.00 b	1.76 ± 0.01 d	1.02 ± 0.00 c	0.17 ± 0.00 a
	25	M3Rha	0.08 ± 0.00 b	0.19 ± 0.02 c	0.19 ± 0.01 c	0.02 ± 0.00 a	0.22 ± 0.01 d	nd
		Sum	1.13 ± 0.01 b	1.82 ± 0.01 e	1.21 ± 0.02 c	2.17 ± 0.01 f	1.66 ± 0.04 d	0.61 ± 0.01 a
		PC (mg/g dm)	37.45 ± 0.65 d	36.62 ± 0.01 c	35.93 ± 0.41 b	39.43 ± 0.18 e	36.65 ± 0.45 c	31.50 ± 0.25 a
Flowers								
*Flavan-3-ols*	5	PT	1.26 ± 0.10 b	1.95 ± 0.06 d	1.46 ± 0.01 c	2.70 ± 0.01 e	0.96 ± 0.00 a	1.52 ± 0.10 c
	6	PD	1.01 ± 0.01 c	1.66 ± 0.05 e	1.15 ± 0.07 d	1.13 ± 0.02 d	0.49 ± 0.00 b	0.36 ± 0.00 a
	11	4EGGlu	3.51 ± 0.01 b	3.22 ± 0.02 a	3.25 ± 0.01 a	7.14 ± 0.01 d	4.13 ± 0.01 c	3.71 ± 0.01 b
		Sum	5.78 ± 0.20 b	6.83 ± 0.10 c	5.87 ± 0.09 b	10.96 ± 0.04 d	5.58 ± 0.02 a	5.58 ± 0.11 a
*Phenolic acids*	13	3PA	3.00 ± 0.00 f	0.79 ± 0.07 b	2.84 ± 0.17 e	1.21 ± 0.02 c	1.84 ± 0.11 d	0.48 ± 0.07 a
	14	4CA	10.72 ± 0.07 d	2.63 ± 0.04 a	9.24 ± 0.23 c	6.70 ± 0.06 b	7.94 ± 0.06 bc	1.80 ± 0.07 a
	15	3CA	15.71 ± 0.00 c	16.73 ± 0.01 cd	15.27 ± 0.16 c	8.65 ± 0.16 a	9.94 ± 0.04 a	13.03 ± 0.04 b
	16	3,4CA	4.60 ± 0.19 c	3.53 ± 0.06 b	5.37 ± 0.03 cd	2.01 ± 0.04 a	2.80 ± 0.06 a	4.31 ± 0.12 bc
		Sum	34.03 ± 0.29 f	23.68 ± 0.19 d	32.72 ± 0.61 e	18.58 ± 0.30 a	22.51 ± 0.28 c	19.62 ± 0.32 b
*Flavonols*	24	A8Glu	1.04 ± 0.01 d	0.69 ± 0.01 b	0.98 ± 0.01 c	0.60 ± 0.02 a	0.55 ± 0.01 a	1.05 ± 0.01 d
	21	Q3Glu	12.08 ± 0.08 c	6.04 ± 0.13 a	12.74 ± 0.07 c	13.06 ± 0.04 d	11.38 ± 0.03 b	14.91 ± 0.18 d
	22	Q3G	1.76 ± 0.07 b	0.88 ± 0.12 a	2.04 ± 0.01 c	1.71 ± 0.00 b	2.26 ± 0.06 d	1.78 ± 0.08 b
	20	Q3R	0.25 ± 0.00 b	0.47 ± 0.00 d	0.20 ± 0.01 b	0.79 ± 0.00 e	0.30 ± 0.01 c	0.06 ± 0.00 a
	26	K3R	1.04 ± 0.02 c	0.66 ± 0.02 b	1.06 ± 0.00 c	1.27 ± 0.01 d	0.40 ± 0.00 a	1.62 ± 0.02 e
	27	K3Rglu	1.01 ± 0.04 b	5.99 ± 0.02 d	1.16 ± 0.03 b	3.77 ± 0.10 c	1.04 ± 0.03 b	0.91 ± 0.00 a
		Sum	17.19 ± 0.24 b	14.71 ± 0.31 a	18.18 ± 0.16 c	21.19 ± 0.19 e	15.94 ± 0.14 a	20.33 ± 0.00 d
		PC (mg/g dm)	57.00 ± 0.64 d	45.22 ± 0.61 b	56.76 ± 0.99 d	50.73 ± 0.66 c	44.03 ± 0.48 a	45.54 ± 0.84 b

Results were expressed as mean (n = 3) ± SD. Statistical significance was analyzed by Duncan’s test. Results marked with different letters (between species) in the same row indicate statistically significant differences (*p* < 0.05). Explanations: *, numbering according to Table S1; PC, sum of phenolic compounds; C3Glu, cyanidin-3-*O*-glucoside; P3R, pelargonidin 3-*O*-rutinoside; C3A cyanidin-3-*O*-arabinoside; P3Glu, Peonidin 3-*O*-glucoside; PT, Procyanidin trimer; PD, Procyanidin dimer; C(+), (+)-catechin; E(−), (−)-epicatechin; Ptet, Procyanidin tetramer; C, Cinchonine; 4EGGlu, 4′′-glucoside of epigallocatechin gallate; QA, Quinic acid; 3PA, 3-*O*-*p*-coumaroylquinic acid; 4CA, 4-*O*-caffeoylquinic acid; 3CA, 3-*O*-caffeoylquinic acid; 3,4CA, 3,4-*O*-dicaffeoylquinic acid; L7Glu, Luteolin 7-C-glucoside; L8Glu, Luteolin 8-C-glucoside; N7Glu, Naringenin 7-*O*-glucoside; Q3Glu, Quercetin 3-*O*-glucoside; Q3G, Quercetin 3-*O*-galactoside; Q3R, Quercetin 3-*O*-rutinoside; A7R, Apigenin 7-*O*-rutinoside; A8Glu, Apigenin 8-C-glucoside; M3Rha, Myricetin 3-*O*-rhamnoside; K3R, Kaempferol 3-*O*-rutinoside; K3Rglu, Kaempferol 3-*O*-rutinoside-7-*O*-glucoside.

**Table 2:** Antioxidant (mmol TE/g; IC_50_, μg/mL), anti-inflammatory (IC_50_, mg/mL), and antidiabetic activity (IC_50_, mg/mL) of hawthorn berries, leaves, and flower preparations.

No.	Species and Morphological Parts	ABTS	CUPRAC	ChP	O_2_˙^−^	OH˙	Trypsin	α-Amylase	α-GlucoSidase
		mmol TE/g		IC_50_, µg/mL			IC_50_, mg/mL		
	Berries								
1	*C. monogyna*	3.81 ± 0.04 bc	4.21 ± 0.04 d	724.11 ± 1.01 g	371.64 ± 0.51 b	112.40 ± 0.05 b	2.25 ± 0.06 f	0.89 ± 0.04 cd	1.28 ± 0.06 c
2	*C. rhipidophylla*	7.03 ± 0.11 h	6.52 ± 0.08 h	608.90 ± 0.83 c	362.44 ± 0.05 a	98.02 ± 0.54 a	1.51 ± 0.04 b	0.79 ± 0.03 c	1.29 ± 0.06 c
3	*C. x subsphaericea*	4.90 ± 0.18 e	5.58 ± 0.10 g	663.35 ± 0.34 f	392.27 ± 0.10 cd	139.25 ± 0.82 d	4.30 ± 0.11 i	0.92 ± 0.04 d	1.41 ± 0.07 d
4	*C. laevigata x rhipidophylla x monogyna*	10.22 ± 0.11 k	7.90 ± 0.09 k	526.44 ± 0.90 a	389.18 ± 0.78 c	112.77 ± 0.38 b	1.58 ± 0.04 b	0.48 ± 0.02 a	0.87 ± 0.04 a
5	*C. x macrocarpa*	9.87 ± 0.10 j	7.24 ± 0.12 j	545.38 ± 1.60 b	401.91 ± 0.36 d	134.72 ± 0.40 c	1.79 ± 0.05 c	0.56 ± 0.03 b	1.01 ± 0.05 b
6	*C. laevigata*	7.88 ± 0.03 i	7.06 ± 0.05 i	1005.62 ± 3.81 o	369.10 ± 0.66 ab	99.11 ± 0.11 a	2.11 ± 0.06 e	1.11 ± 0.05 ef	1.57 ± 0.08 de
	Leaves								
1	*C. monogyna*	3.60 ± 0.03 b	4.21 ± 0.03 d	654.50 ± 0.31 e	497.80 ± 0.30 i	232.42 ± 0.33 h	2.00 ± 0.05 d	1.16 ± 0.05 f	1.91 ± 0.10 f
2	*C. rhipidophylla*	4.55 ± 0.11 d	4.34 ± 0.09 d	802.31 ± 0.16 k	503.41 ± 0.41 j	224.91 ± 0.72 g	2.30 ± 0.06 fg	1.20 ± 0.05 g	1.95 ± 0.10 fg
3	*C. x subsphaericea*	4.92 ± 0.14 e	4.66 ± 0.13 e	725.03 ± 0.60 g	413.13 ± 0.34 e	165.60 ± 0.29 e	2.01 ± 0.05 d	1.33 ± 0.06 h	2.03 ± 0.10 g
4	*C. laevigata x rhipidophylla x monogyna*	5.67 ± 0.06 g	5.48 ± 0.06 g	628.97 ± 1.10 d	457.94 ± 0.07 g	188.28 ± 0.54 f	1.58 ± 0.04 b	0.80 ± 0.04 c	1.27 ± 0.06 c
5	*C. x macrocarpa*	5.59 ± 0.03 g	5.12 ± 0.04 f	655.29 ± 0.07 e	457.42 ± 0.44 g	187.63 ± 0.07 f	1.84 ± 0.05 c	1.00 ± 0.04 e	1.88 ± 0.09 f
6	*C. laevigata*	5.30 ± 0.08 f	5.10 ± 0.04 f	966.83 ± 0.84 n	410.39 ± 0.12 e	163.75 ± 0.83 e	3.85 ± 0.10 h	1.37 ± 0.06 h	2.14 ± 0.11 h
	Flowers								
1	*C. monogyna*	3.81 ± 0.09 bc	3.71 ± 0.12 b	781.63 ± 0.43 i	462.22 ± 0.13 h	273.20 ± 0.30 i	0.69 ± 0.02 a	0.83 ± 0.04 c	1.33 ± 0.07 c
2	*C. rhipidophylla*	3.84 ± 0.11 bc	3.96 ± 0.04 c	827.76 ± 0.08 l	458.74 ± 0.53 g	310.66 ± 0.89 j	2.36 ± 0.06 g	1.22 ± 0.05 g	2.51 ± 0.13 j
3	*C. x subsphaericea*	3.92 ± 0.13 c	3.58 ± 0.07 a	740.37 ± 0.18 h	444.11 ± 0.16 f	270.12 ± 1.45 i	2.25 ± 0.06 f	0.87 ± 0.04 cd	1.85 ± 0.09 f
4	*C. laevigata x rhipidophylla x monogyna*	4.18 ± 0.06 c	3.80 ± 0.01 bc	791.13 ± 0.06 j	463.43 ± 0.48 h	309.60 ± 0.03 k	1.56 ± 0.04 b	0.95 ± 0.04 d	1.64 ± 0.08 e
5	*C. x macrocarpa*	3.34 ± 0.07 a	3.63 ± 0.05 a	903.03 ± 0.11 m	580.06 ± 0.07 k	517.42 ± 0.70 l	2.31 ± 0.06 fg	1.45 ± 0.06 i	2.51 ± 0.13 j
6	*C. laevigata*	3.62 ± 0.03 b	3.51 ± 0.04 a	1012.57 ± 0.35 o	614.74 ± 0.41 l	534.61 ± 1.05 m	4.95 ± 0.13 j	1.06 ± 0.05 e	2.29 ± 0.12 i
	Mean								
	Berries	7.33 ± 1.83 C	6.44 ± 2.10 C	678.93 ± 9.60 A	381.10 ± 19.13 A	116.02 ± 15.50 A	2.26 ± 0.96 A	0.79 ± 0.22 A	1.24 ± 0.25 A
	Leaves	4.94 ± 2.37 B	4.86 ± 0.93 B	738.83 ± 16.28 B	456.63 ± 10.35 B	193.73 ± 22.26 B	2.26 ± 0.77 A	1.14 ± 0.21 C	1.86 ± 0.30 B
	Flowers	3.88 ± 1.90 A	3.71 ± 0.71 A	842.71 ± 12.04 C	503.92 ± 8.80 C	369.33 ± 8.11 C	2.35 ± 1.34 B	1.07 ± 0.23 B	2.02 ± 0.47 C

Results are expressed as mean (n = 3) ± SD. The values in the columns marked with different letters (lowercase letters, between species and morphological parts; uppercase letters, between means for morphological parts) indicate statistically significant differences (*p* < 0.05).

**Table 3:** Cytotoxic activity (IC_50_, μg/mL) of hawthorn berries, leaves, and flower preparations.

No.	Species and Morphological Parts	AGS	Caco-2	Dld-1	Sk-mel-28	Mcf-7	CCD841CoN
		IC_50_, μg/mL					
	Berries						
1	*C. monogyna*	265.60 ± 11.31 j	189.71 ± 5.62 e	288.83 ± 11.75 h	402.57 ± 8.34 i	252.84 ± 1.63 f	>500
2	*C. rhipidophylla*	111.34 ± 4.89 a	98.48 ± 2.96 a	165.31 ± 6.46 ab	350.74 ± 10.77 fg	127.05 ± 0.87 a	357.35 ± 13.77 a
3	*C. x subsphaericea*	280.12 ± 10.22 k	299.95 ± 8.93 i	313.04 ± 11.01 i	440.72 ± 3.37 j	256.25 ± 7.79 f	464.12 ± 9.30 h
4	*C. laevigata x rhipidophylla x monogyna*	157.06 ± 6.75 bc	230.31 ± 6.85 g	224.51 ± 7.91 d	250.44 ± 7.92 c	191.63 ± 5.20 d	457.96 ± 3.05 h
5	*C. x macrocarpa*	199.11 ± 8.74 e	127.64 ± 3.89 b	158.40 ± 3.24 a	295.62 ± 9.04 d	173.59 ± 3.63 c	408.92 ± 5.28 e
6	*C. laevigata*	117.03 ± 4.78 a	106.20 ± 9.05 a	230.29 ± 9.31 f	370.00 ± 1.91 h	135.11 ± 2.97 ab	>500
	Leaves						
1	*C. monogyna*	207.43 ± 8.90 ef	217.92 ± 6.54 g	334.02 ± 8.45 jk	458.24 ± 5.52 k	214.16 ± 6.80 e	377.11 ± 8.17 bc
2	*C. rhipidophylla*	252.22 ± 1.69 i	329.13 ± 9.85 j	367.69 ± 10.36 l	359.52 ± 11.00 gh	259.29 ± 2.90 f	>500
3	*C. x subsphaericea*	212.39 ± 9.13 fg	274.69 ± 8.25 h	398.66 ± 8.14 m	313.65 ± 9.62 e	282.25 ± 3.81 g	>500
4	*C. laevigata x rhipidophylla x monogyna*	160.88 ± 6.90 c	168.12 ± 2.39 d	253.52 ± 9.92 g	310.39 ± 6.46 e	184.72 ± 11.54 d	387.62 ± 10.55 cd
5	*C. x macrocarpa*	186.75 ± 8.03 d	189.61 ± 5.68 e	229.21 ± 9.31 f	489.43 ± 3.65 m	213.27 ± 7.13 e	488.83 ± 7.98
6	*C. laevigata*	329.41 ± 2.98 m	365.03 ± 10.91 k	421.90 ± 0.90 n	>500	338.52 ± 3.92 h	>500
	Flowers						
1	*C. monogyna*	221.72 ± 3.97 gh	199.84 ± 6.05 ef	185.12 ± 7.55 cd	206.20 ± 6.32 a	251.92 ± 10.33 f	369.93 ± 8.51 b
2	*C. rhipidophylla*	153.53 ± 6.67 bc	156.72 ± 4.73 d	324.44 ± 8.97 ij	339.40 ± 10.41 f	189.92 ± 1.35 d	427.86 ± 2.66 g
3	*C. x subsphaericea*	178.71 ± 3.00 d	160.41 ± 11.21 d	220.38 ± 8.99 e	345.17 ± 7.06 f	143.71 ± 4.87 b	391.43 ± 7.96 d
4	*C. laevigata x rhipidophylla x monogyna*	146.11 ± 6.24 b	139.77 ± 2.17 c	178.87 ± 6.96 bc	228.83 ± 7.08 b	138.18 ± 4.24 b	423.12 ± 11.91 f
5	*C. x macrocarpa*	297.13 ± 12.72 l	204.35 ± 6.11 f	395.18 ± 5.41 m	>500	342.04 ± 9.83 h	>500
6	*C. laevigata*	227.28 ± 3.65 h	265.92 ± 7.90 h	339.21 ± 1.74 k	473.62 ± 2.84 l	392.92 ± 4.38 i	>500
	Mean						
	Berries	188.41 ± 72.80 A	175.43 ± 79.71 A	230.13 ± 62.86 A	368.28 ± 50.04 B	189.49 ± 55.89 A	448.02 ± 55.71 B
	Leaves	224.87 ± 59.52 C	257.48 ± 78.93 C	334.16 ± 78.11 C	407.12 ± 90.27 C	248.66 ± 56.32 B	458.92 ± 59.68 C
	Flowers	204.14 ± 56.77 B	187.83 ± 45.98 B	273.83 ± 90.95 B	349.55 ± 122.16 A	243.13 ± 105.91 B	435.47 ± 54.40 A

Results are expressed as mean (n = 9) ± SD. The values in the columns marked with different letters (lowercase letters, between species and morphological parts; uppercase letters, between means for morphological parts) indicate statistically significant differences (*p* < 0.05).

## Data Availability

Data are available from the authors.

## Supplementary Materials

The following supporting information can be downloaded at: https://www.mdpi.com/article/10.3390/molecules29235786/s1, Table S1. Polyphenolic compounds identified by the UPLC-PDA-ESI-MS method in hawthorn berry, leaves, and flower preparations. Figure S1. UPLC chromatogram of polyphenolic compounds obtained for hawthorn fruit preparation (C4). Figure S2. UPLC chromatogram of polyphenolic compounds obtained for a hawthorn fruit preparation (C4). Figure S3. UPLC chromatogram of polyphenolic compounds obtained for a hawthorn leaf preparation (C4). Figure S4. UPLC chromatogram of polyphenolic compounds obtained for a hawthorn flower preparation (C4).
