# Supplementary material for: Berries, Leaves, and Flowers of Six Hawthorn Species (Crataegus L.) as a Source of Compounds with Nutraceutical Potential
Source: Molecules. 2024 Dec 7;29(23):5786. doi: 10.3390/molecules29235786 (PMC11643722; doi:10.3390/molecules29235786)
Supplement: Supplementary file 1 [file molecules-29-05786-s001.zip › molecules-3353018-supplementary.pdf]

molecules

MDPI

Article

Berries, Leaves, and Flowers of Six Hawthorn Species (*Crataegus* L.) as a Source of Compounds with Nutraceutical Potential

Natalia Żurek [1], Michał Świeca [2] and Ireneusz Tomasz Kapusta [1,*]

[1] Department of Food Technology and Human Nutrition, College of Natural Sciences, University of Rzeszow, 4 Zelwerowicza St., 35-601 Rzeszow, Poland; nzurek@ur.edu.pl
[2] Department of Food Chemistry and Biochemistry, University of Life Sciences in Lublin, 8 Skromna St., 20-704 Lublin, Poland; michal.swieca@up.lublin.pl
* Correspondence: ikapusta@ur.edu.pl

Content:

Table S1. Polyphenolic compounds identified by the UPLC-PDA-ESI-MS method in hawthorn berry, leaves, and flower preparations.
Figure S1. UPLC chromatogram of polyphenolic compounds obtained for hawthorn fruit preparation (C4).
Figure S2. UPLC chromatogram of polyphenolic compounds obtained for a hawthorn fruit preparation (C4).
Figure S3. UPLC chromatogram of polyphenolic compounds obtained for a hawthorn leaf preparation (C4).
Figure S4. UPLC chromatogram of polyphenolic compounds obtained for a hawthorn flower preparation (C4).

Table S1. Polyphenolic compounds identified by the UPLC-PDA-ESI-MS method in hawthorn berry, leaves, and flower preparations.

No	Identified compound	λ_{max}	[M-H] *m/z*		Morphological parts		
		nm	MS	MS/MS	B	L	F
Anthocyanins							
**1**	Cyanidin 3-*O*-glucoside	278, 514	449^{+}	287	+		
**2**	Pelargonidin 3-*O*-rutinoside	279, 517	579^{+}	271, 433	+		
**3**	Cyanidin 3-*O*-arabinoside	274, 510	419^{+}	287	+		
**4**	Peonidin 3-*O*-glucoside	278, 516	463^{+}	301	+		
Flavan-3-ols							
**5**	Procyanidin trimer (type B)	278	865	289	+	+	+
**6**	Procyanidin dimer (type B)	279	577	289	+	+	+
**7**	(+)-catechin	281	289	-	+		
**8**	(-)-epicatechin	274	289	-		+	
**9**	Procyanidin tetramer (type B)	278	1442	720, 577, 289		+	
**10**	Cinchonine	279, 377	451	341, 315	+		
**11**	4''-glucoside of epigallocatechin gallate	268, 377	619	169, 305, 301	+	+	+
Phenolic acids							
**12**	Quinic acid	274	191	173	+		
**13**	3-*O*-*p*-coumaroylquinic acid	309	337	163, 119	+	+	+
**14**	4-*O*-caffeoylquinic acid	299sh, 324	353	191, 173	+	+	+
**15**	3-*O*-caffeoylquinic acid	299sh, 321	353	191, 173	+	+	+
**16**	3,4-*O*-dicaffeoylquinic acid	299sh, 327	515	353		+	+
Flavonols							
**17**	Luteolin 8-*C*-glucoside	270, 350	447	285	+		
**18**	Luteolin 7-*O*-glucoside	265, 352	447	285, 249		+	
**19**	Naringenin 7-*O*-glucoside	272, 353	433	271	+		
**20**	Quercetin 3-*O*-rutinoside	289sh, 353	609	301, 300, 271			+
**21**	Quercetin 3-*O*-glucoside	255, 355	463	301	+	+	+
**22**	Quercetin 3-*O*-galactoside	255, 352	463	301		+	+
**23**	Apigenin 7-*O*-rutinoside	266, 338	577	296, 112	+		
**24**	Apigenin 8-*C*-glucoside	266, 338	431	311, 341, 289	+		+
**25**	Myricetin 3-*O*-rhamnoside	281	463	317		+	
**26**	Kaempferol 3-*O*-rutinoside	345	593	285			+
**27**	Kaempferol 3-*O*-rutinoside-7-*O*-glucoside	288, 347	725	547, 285			+

Aberrations: UV-Vis, ultraviolet-visible; $[M\text{-}H]^{-}$, negative ion values; *m/z*, mass-to-charge ratio; B, berries; L, leaves; F, flowers; +, compound identified.

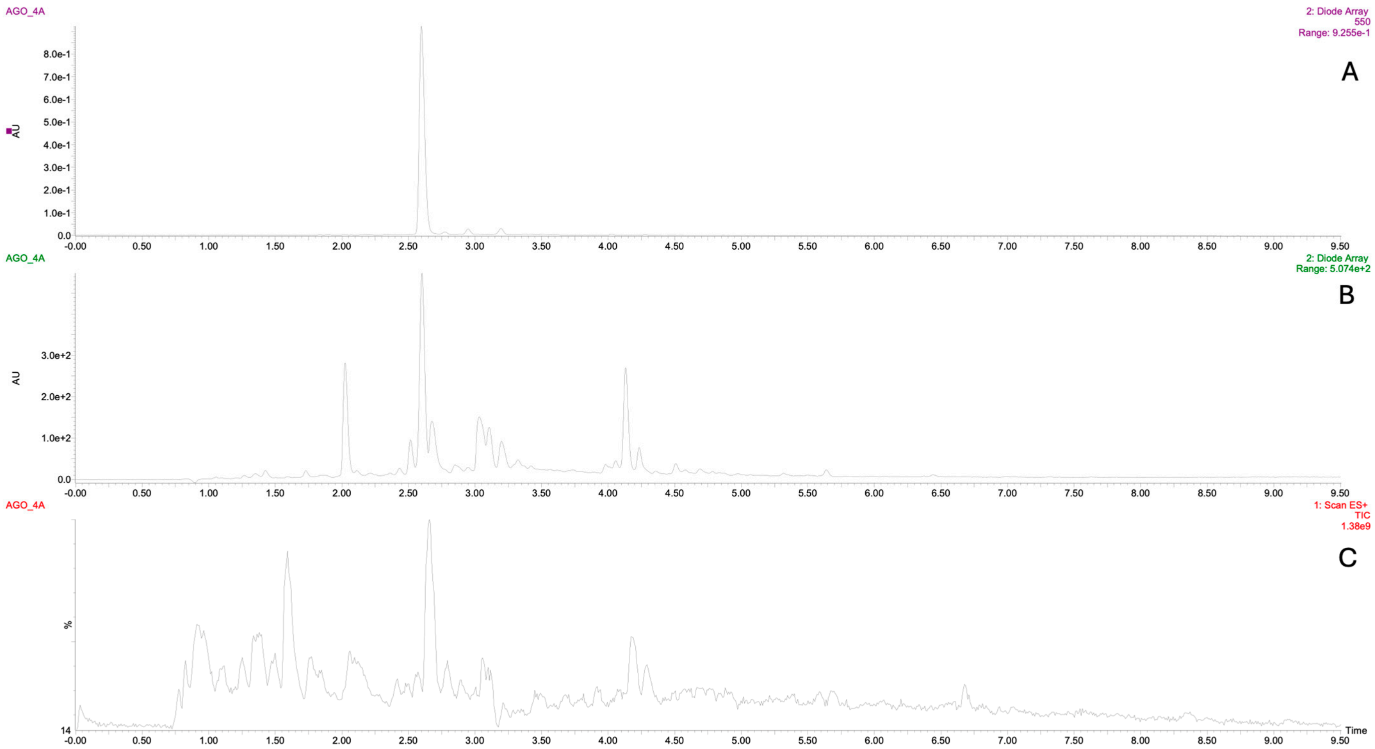

Figure S1. UPLC chromatogram of polyphenolic compounds obtained for hawthorn fruit preparation (C4). A, PDA chromatogram at 520 nm for visualization of anthocyanins; B, PDA chromatogram at 350 nm for other phenolic compounds; C, total ion current.

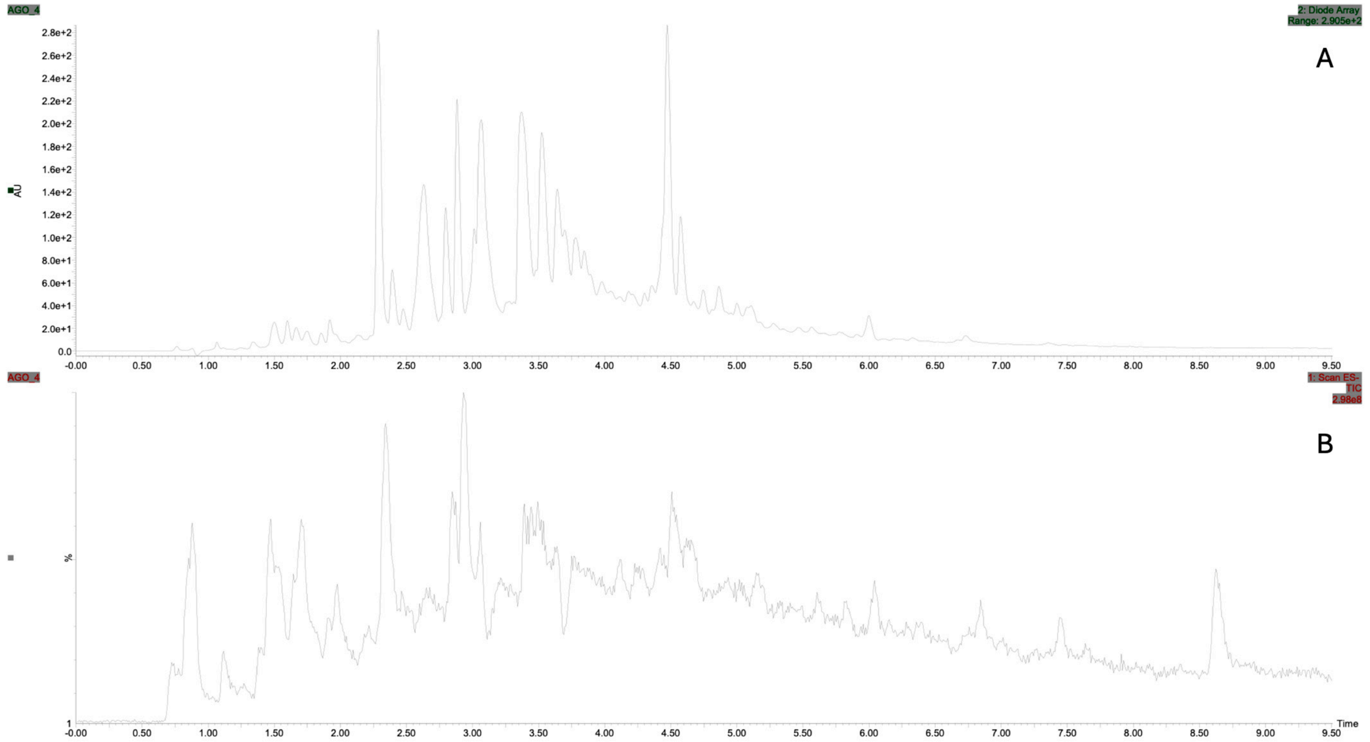

Figure S2. UPLC chromatogram of polyphenolic compounds obtained for a hawthorn fruit preparation (C4). A, PDA chromatogram at 350 nm for polyphenolic compounds; B, total ion current.

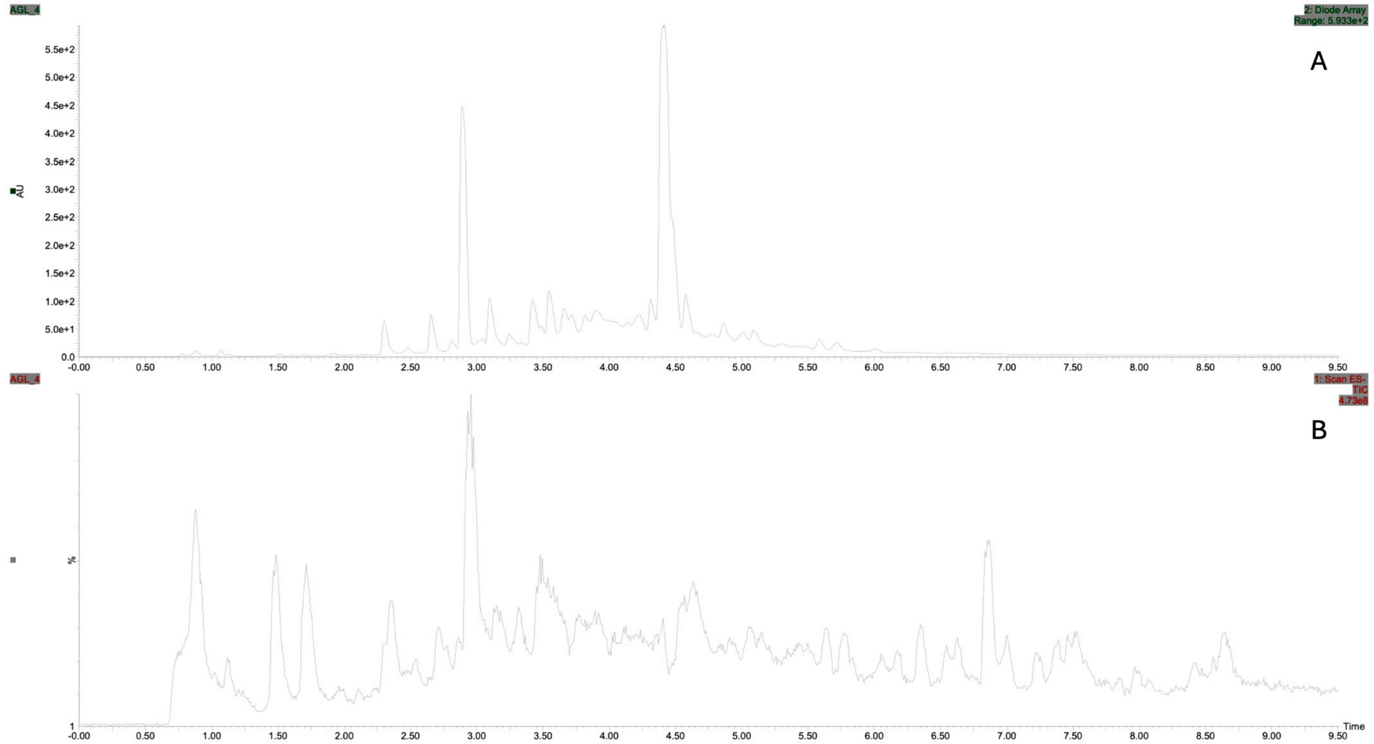

Figure S3. UPLC chromatogram of polyphenolic compounds obtained for a hawthorn leaf preparation (C4). A, PDA chromatogram at 350 nm for polyphenolic compounds; B, total ion current.

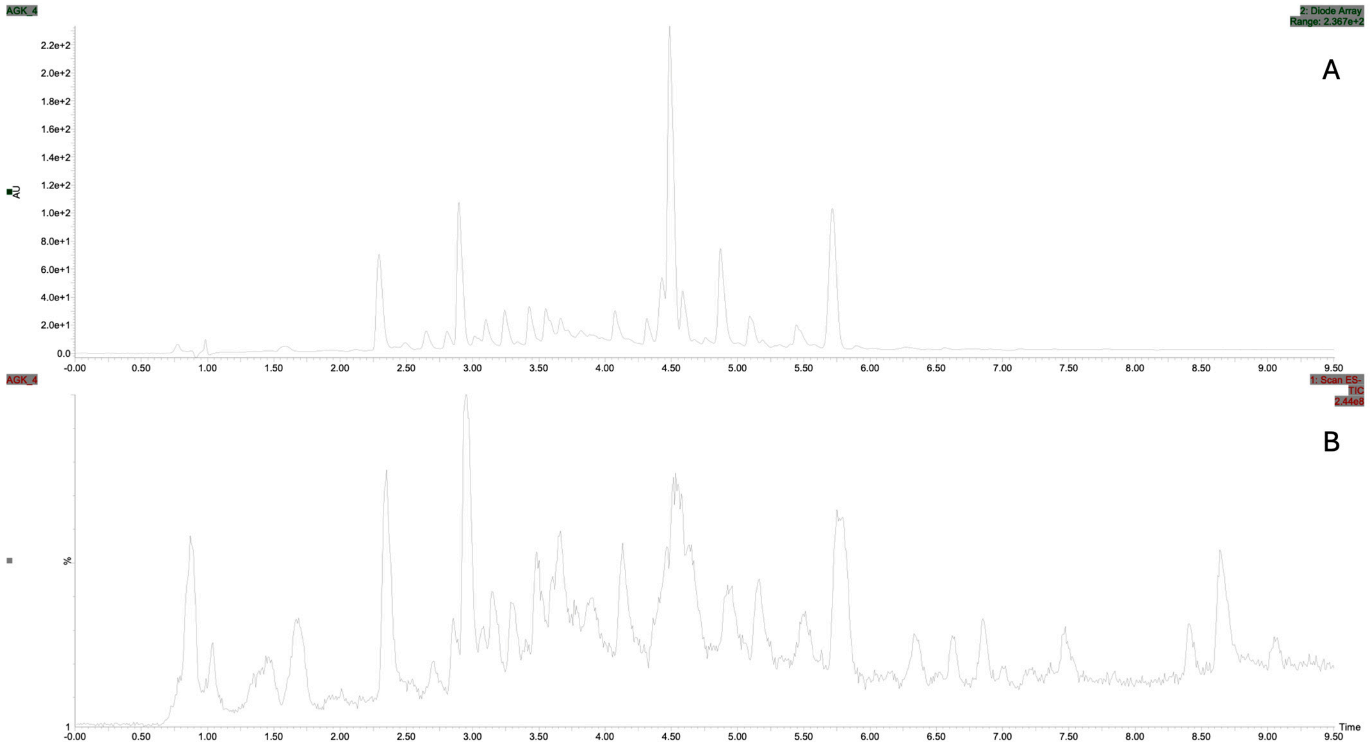

Figure S4. UPLC chromatogram of polyphenolic compounds obtained for a hawthorn flower preparation (C4). A, PDA chromatogram at 350 nm for polyphenolic compounds; B, total ion current.
